# THE EFFECTS OF LATE-LIFE RELOCATION ON ANXIETY AND PERSONALITY TRAITS: EVIDENCE FROM CHINA AND EUROPE

**DOI:** 10.1093/geroni/igad104.2154

**Published:** 2023-12-21

**Authors:** Yang Sun, Cornelia Wrzus, Shao-bo Lv

**Affiliations:** Ruprecht Karl University of Heidelber, Heidelberg, Baden-Wurttemberg, Germany; Heidelberg University, Heidelberg, Baden-Wurttemberg, Germany; North China University of Technology, Tangshan, Hebei, China (People’s Republic)

## Abstract

Later-life relocation has been identified as an important life event that affects the living environment, social relationships, and daily routines of older adults with subsequent effects on mental health and personality. We explored the impact of relocation on anxiety and personality traits in older adults from China and Europe in two separate studies., In study 1, we administered the questionnaire to 301 Chinese older adults (Mage=69.51, SD=5.02) who experienced involuntary rural-urban relocation, with 61.8% of them reported severe to extremely severe anxiety. Excessive reassurance-seeking and concomitant negative information bias were processes that contributed to increased anxiety among relocated older adults, which was particularly detrimental for those adults who were otherwise resilient. In study 2, we investigated the differences in personality traits between older adults who moved to nursing homes and those who lived in private housing using data from Wave 7 of the European Health, Ageing and Retirement Survey (SHARE). Reports from a total of 4829 older people living in nursing homes (NNH= 360) and private housing (NPH=4469) were included in the finally analysis. Our results showed that older people who relocated to nursing homes had lower extraversion and conscientiousness, and higher neuroticism. Additionally, older adults’ age, gender, health problems, physical activity, community size, and national socio-economic status partly explained personality differences between older adults who relocated and who stayed in their homes. Our findings underscore the importance and necessity of studying advantageous and detrimental effects of late-life relocation, and provide new directions and ideas for future research.

